# Evaluating Sleep Apnea: In‐Lab Versus at‐Home Recording Time and the Impact of Positional Disease

**DOI:** 10.1002/lary.70517

**Published:** 2026-03-20

**Authors:** Raquel Chartuni Pereira Teixeira, Michel Burihan Cahali

**Affiliations:** ^1^ Department of Otolaryngology, Hospital das Clinicas HCFMUSP, Faculdade de Medicina da Universidade de São Paulo Universidade de Sao Paulo Sao Paulo Brazil

**Keywords:** diagnosis, home sleep apnea test, obstructive sleep apnea, polysomnography, recording time

## Abstract

**Objectives:**

To determine the minimum required recording time (RRT) for in‐lab Type 1 polysomnography (PSG1) and home sleep apnea test with Type 3 portable monitoring (HSAT) that consistently reflects the full‐night apnea‐hypopnea index (AHI) in patients with and without positional obstructive sleep apnea (OSA).

**Methods:**

This prospective randomized crossover trial with 54 consecutive patients with suspected OSA involved PSG1 and HSAT no more than 7 days apart (108 exams). Each test was analyzed in 60‐min intervals. Minimum RRT was the point the test achieved and maintained an AHI difference < 5 events/h from end‐test AHI. Patients were grouped into non‐positional and positional OSA, and by severity subgroups.

**Results:**

Minimum RRT in PSG1/HSAT was 4 h/2 h for the entire cohort, 5 h/3 h for non‐positional OSA and 6 h/2 h for positional OSA, respectively. Patients with AHI < 15 events/h showed minimum RRT of 2 h in both exams and a clinically irrelevant AHI difference (below 5 events/h) between PSG1 and HSAT after 4 h. Conversely, patients with AHI ≥ 15 events/h presented minimum RRT of 6 h/4 h in PSG1/HSAT, respectively. By adopting an AHI difference threshold of < 10 events/h, minimum RRT in this latter group was 4 h/2 h in PSG1/HSAT.

**Conclusion:**

To account for the influence of positional and non‐positional OSA, the minimum required recording times for PSG1 and HSAT that safely reflect the end‐test AHI are 6 and 3 h, respectively. Unlike HSAT, positional OSA hinders PSG1's ability to adequately anticipate end‐test AHI until the exam is nearly complete.

**Level of Evidence:**

2.

## Graphical abstract

We evaluated the minimum recording time needed for in‐lab polysomnography (PSG1) and home sleep apnea test Type 3 portable monitoring (HSAT) to accurately reflect the full‐night apnea–hypopnea index (AHI) in patients with suspected obstructive sleep apnea (OSA). To account for the presence of positional and non‐positional OSA, the minimum required recording times for PSG1 and HSAT that safely reflect the end‐test AHI are 6 and 3 h, respectively.

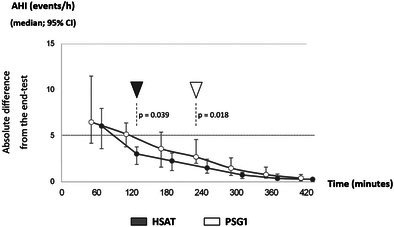

## 1 Introduction

Estimates show that approximately 1 billion people have obstructive sleep apnea (OSA) when considering an apnea‐hypopnea index (AHI) greater than or equal to 5 events/h of sleep [1]. Currently, diagnosis depends on polysomnography [2], with the gold standard being supervised laboratory polysomnography (Type 1, PSG1). PSG1 has been considered a technically complex test of high cost and is sometimes not available [3]. An alternative is home sleep apnea test with cardiorespiratory portable monitoring (Type 3, HSAT), which represents a cheaper and more accessible technology [4]. HSAT, however, does not usually identify wakefulness and sleep states and is currently recommended in patients with a high pretest probability of OSA who do not present relevant comorbidities.

A question frequently asked by patients and physicians is about the time needed for the sleep test to obtain a reliable result for OSA. In HSAT, there is a risk of loss of information due to a sensor falling or by the voluntary interruption of the exam. American Academy of Sleep Medicine (AASM) officially recommends a minimum recording time of 4 h with suitable sensors for validating HSAT. The level of evidence for this official recommendation is considered very low, as there is not enough data to indicate that HSAT recordings shorter than 4 h would show significantly different results compared to longer recordings [4]. Besides the cost for any health system, the need to repeat a sleep test due to a record of less than 4 h can be an avoidable element of stress in the doctor–patient relationship.

Time spent in the supine position can significantly change the AHI, which is particularly relevant in patients with mild to moderate OSA [5]. The collapsibility of the upper airway is increased in the supine position due to gravitational effect and changes in size of the upper airway [6] [7]. Patients with marked increase of AHI in supine, called positional OSA (POSA), have a significant reduction in lung volume at the end of expiration, during sleep, when changing from the lateral to supine position, which increases the collapsibility of the upper airway [8]. In HSAT, performed in a home environment (less “hostile”) and without the head sensors used to monitor sleep, patients may feel free to move and, thus, spend less time in supine, which appears to reduce OSA severity, particularly in patients with more severe OSA [9]. The influence of body position and POSA on the minimum required recording time (RRT) for sleep tests is largely ignored and may bear a huge impact on policies for repeating a HSAT or performing split night PSG1. We aimed to verify the influence of POSA on the minimum RRT for PSG1 and HSAT to accurately reflect full‐night test outcomes. Our hypothesis was that patients with POSA, compared with patients without POSA, would require longer minimum monitoring time in sleep tests to accomplish that task.

## 2 Methods

### 2.1 Study Design and Participants

We conducted a prospective, single center, randomized, crossover examiner‐blinded trial between September 2018 (first patient enrollment) and July 2021 (last patient completed). Patients aged > 18 years referred for polysomnography who agreed to undergo PSG1 and HSAT within a maximum interval of 7 days were consecutively evaluated. Patients must relate at least one OSA complaint (habitual snoring, observed apneas, choking sensation, drowsiness or nonrestorative sleep). We only analyzed the results of patients who spent at least 30 min each in supine and nonsupine positions during sleep in PSG1. We excluded patients with significant cardiopulmonary or neuromuscular diseases, previous stroke, chronic use of opioids, severe insomnia, restless legs syndrome, Rapid eye movement sleep behavior disorder and other parasomnias, and inability to understand the instructions for performing the HSAT.

The trial protocol was approved by our institutional review board (No. 2,954,801) and registered in the National Unified Database for Human Research: Plataforma Brasil (No. CAAE 83077618.9.3001.0065, URL https://plataformabrasil.saude.gov.br/login.jsf). Written informed consent was obtained from each patient.

**FIGURE 1:**
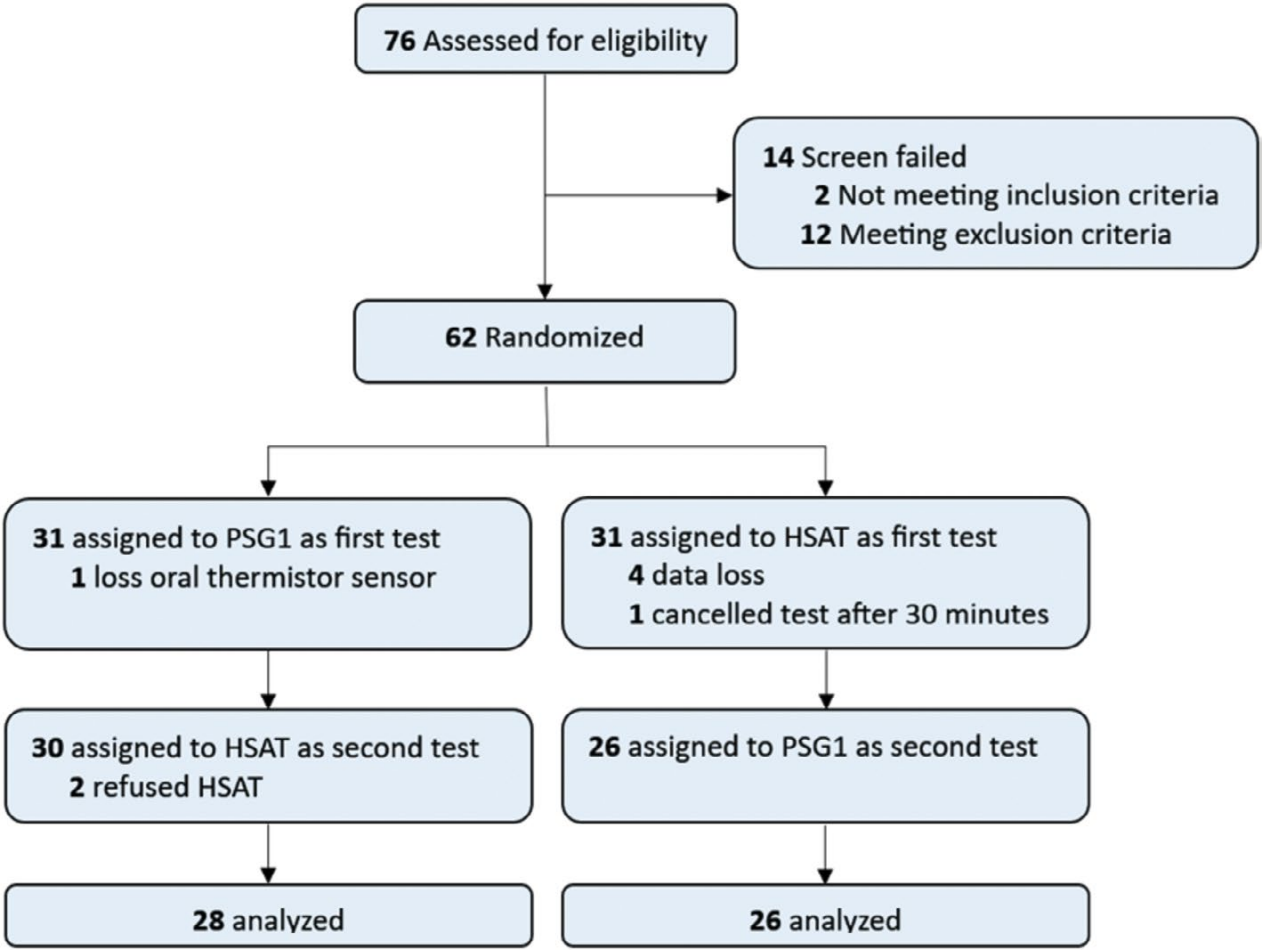
Diagram for study enrollment, allocation and follow‐up. PSG1 = in‐lab Type 1 polysomnography; HSAT = home sleep apnea test with cardiorespiratory Type 3 portable monitoring. [Color figure can be viewed in the online issue, which is available at www.laryngoscope.com]

Figure 1 presents a diagram of patient screening and enrollment. After screening 76 patients, 62 were randomized 1:1 for the sequence of the sleep tests (PSG1 or HSAT) using the RandomNumberGenerator software from GraphPad (San Diego, CA, USA).

### 2.2 Procedures

For each patient, we evaluated the body mass index (BMI, kg/m^2^) [10], neck circumference (cm) [11] and daytime sleepiness using the Epworth Sleepiness Scale [12]. PSG1 was performed in a sleep lab using the iBlue 64 equipment (Icelera, Vargem Grande Paulista, Brazil) with six electroencephalogram channels, two oculogram channels, a nasal cannula and an oronasal thermistor, chest and abdominal plethysmographic straps for respiratory effort, pulse oximeter, two chest electrodes for cardiac monitoring, thoracic body position sensor, two electrodes on the anterior tibial region and three electrodes in the mental region. HSAT was performed at the patient's home, unsupervised, using Alice PDX equipment (Respironics, Murraysville, PA, USA), with nasal cannula and oronasal thermistor, chest and abdominal plethysmography straps, pulse oximetry and a thoracic body position sensor. All tests were done during a full night of sleep. We analyzed patients that completed both sleep tests, in a random order, no more than 7 days apart.

Apneas were defined as a reduction of at least 90% in the oronasal thermistor amplitude for 10 s or more in both exams. If this signal failed, the nasal cannula was used as an alternative. In PSG1, hypopneas were scored by a reduction of at least 30% in the nasal cannula signal, lasting at least 10 s associated with a reduction of at least 3% in oxyhemoglobin saturation or an arousal. In HSAT, hypopneas were defined as a decrease of at least 30% in the nasal cannula signal associated with a decrease of at least 3% in oxyhemoglobin saturation, for at least 10 s. In the case of malfunctioning or poor signal of the nasal cannula, the oronasal thermistor signal was used [13].

All sleep tests were analyzed by a single examiner, blinded to patient's identity. PSG1 and HSAT were divided into 60‐min intervals from the beginning of the recording, generating the timeframes: 60, 120, 180, 240, 300, 360, 420 min, and whole night exam. For each of these intervals, we calculated the AHI (number of apneas and hypopneas per hour of total sleep time for PSG1, or per hour of recording time for HSAT), the supine AHI (AHI in supine position) and nonsupine AHI (AHI in nonsupine position) in both tests. In addition, we analyzed the oxygen desaturation index (ODI), which refers to the average number of desaturation episodes occurring per hour, where desaturation episodes are defined as a decrease in the mean oxygen saturation of ≥ 3% (over the last 120 s) that lasts for at least 10 s.

According to the whole night outcome in PSG1, we grouped patients into positional (POSA) and non‐positional OSA (non‐POSA). To be included in the POSA group, patients had to meet the Cartwright's criterion, that is, a supine AHI of at least twice as high as the nonsupine AHI [14], and all included patients spent at least 30 min each in supine and nonsupine positions during sleep in PSG1. For further comparisons, we also segregated the severity subgroups according to the AHI in PSG1: simple snorers and mild OSA (AHI < 15 events/h) or moderate‐to‐severe OSA (AHI ≥ 15 events/h).

We defined the minimum RRT as the point when the test significantly achieved and subsequently maintained an AHI difference of less than 5 events/h from the full‐night AHI. This threshold was chosen because 5 events/h is the maximum possible difference between an OSA‐free state and a positive OSA diagnosis. We analyzed the minimum RRT for the entire group, for POSA and non‐POSA patients, and for the OSA severity subgroups in PSG1 and HSAT.

### 2.3 Statistical Analysis

To assess the normality of the variables, we used the Kolmogorov–Smirnov test. Continuous data were expressed as mean and standard deviation (SD) or median, interquartile range (IQR) and 95% confidence intervals (95% CI) where appropriate. To compare the diagnostic variables between PSG1 and HSAT, we used the Wilcoxon test. We evaluated the agreement in AHI between PSG1 and HSAT estimating the intraclass correlation coefficient (ICC) for a mixed model with two factors and absolute agreement. Furthermore, the absolute differences in AHI across the night between PSG1 and HSAT were compared across severity subgroups using the Mann–Whitney U test. For the entire cohort, for POSA and non‐POSA patients, and for the OSA severity subgroups, the absolute difference from the end‐test AHI was calculated for each 60‐min interval across the night. A one‐sided Wilcoxon test was then applied to establish the minimum RRT. All statistical analyses were performed using IBM SPSS Statistics v.29.0.2 software for Windows, with a significance level of 5% [15].

## 3 Results

**FIGURE 2:**
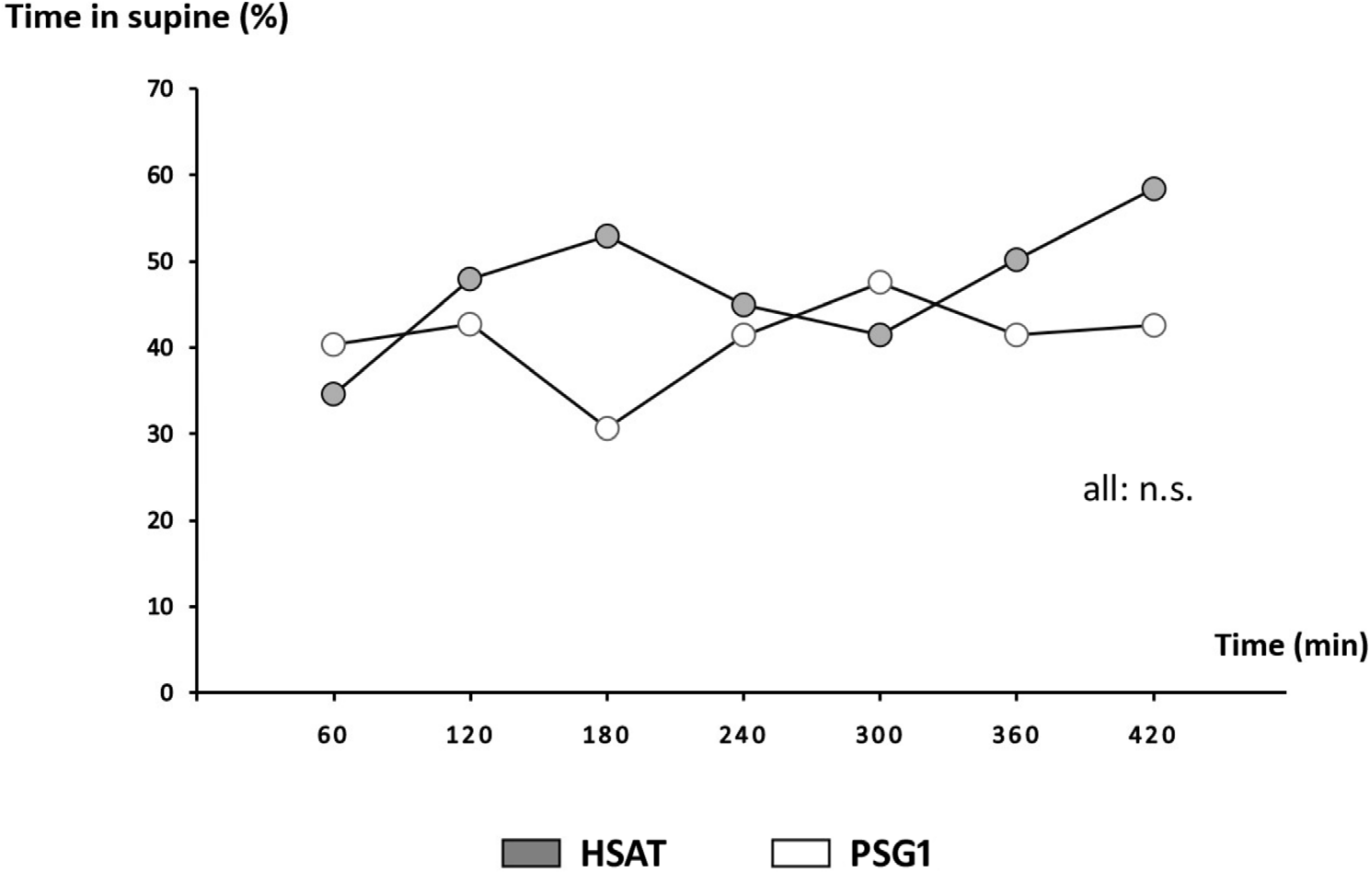
Average supine time in each 1‐h block of the exam (non‐cumulative) for Type 1 polysomnography (PSG1; % supine of sleep time) and Type 3 home sleep apnea test (HSAT; % supine of recording time).

Of the 62 patients included, 31 were randomized to start at each study arm and 54 completed both sleep tests, generating 108 exams that were included in the analysis. No participants were excluded based on spending less than 30 min in either the supine or non‐supine position. Twenty‐eight completed the arm that started with PSG1 and 26 completed the arm that started with HSAT (Figure 1). Patient demographics and clinical characteristics are presented in Table 1. PSG1 exhibited higher AHI, while ODI showed no significant difference between PSG1 and HSAT. Similarly, total supine time, measured in 1‐h intervals across the night, did not significantly differ between PSG1 and HSAT at any interval (Figure 2). Across the night, supine time varied from 6.0% to 92.0% of total sleep time (mean 40.7%; median 37.3%) for PSG1 and from 7.3% to 91.0% of total recording time (mean 40.3%; median 37.7%) for HSAT. According to PSG1, there were 6 subjects with AHI < 5 events/h (simple snorers); 15 with 5 ≤ AHI < 15 events/h (mild OSA); 14 with 15 ≤ AHI ≤ 30 events/h (moderate OSA) and 19 with AHI > 30 events/h (severe OSA).

**TABLE 1:** Patient demographics and clinical characteristics.

Characteristic	PSG1	HSAT	*p*
Age, mean ± SD, years	42.7 ± 13.6		
Body mass index, mean ± SD, kg/m^2^	29.0 ± 3.9		
Neck circumference, mean ± SD, cm	37.8 ± 3.3		
Epworth sleepiness scale, mean ± SD	10.0 ± 4.6		
Male/Female sex, %	75.9/24.1		
AHI, median (IQR)	20.6 (7.3–38.0)	12.8 (4.9–34.9)	0.001*
ODI, median (IQR)	8.3 (1.9–23.3)	8.8 (1.9–26.3)	0.83
T90, mean ± SD, minutes	16.3 ± 36.4	9.7 ± 17.9	0.12
TST or TRT, mean ± SD, minutes^a^	341.0 ± 87.8	412.2 ± 44.8	0.048*
Time in supine, mean ± SD, minutes	215.0 ± 150.4	226.8 ± 146.4	0.28
Sleep efficiency, mean ± SD, %	84.3 ± 10.3	N/A	

Abbreviations: AHI, apnea‐hypopnea index; HSAT, home sleep apnea test with cardiorespiratory Type 3 portable monitoring; IQR, interquartile range; N/A, not applicable; ODI, oxygen desaturation index; PSG1, in‐lab Type 1 polysomnography; SD, standard deviation; TRT, total recording time; TST, total sleep time; T90, total time with oxygen saturation below 90%.

^a^ TST for PSG1 and TRT for HSAT.

* *p* < 0.05 (Wilcoxon test).

The sequence of the sleep tests did not seem to have interfered with OSA evaluation. The mean (median) [minimum—maximum] absolute difference of the AHI between PSG1 and HSAT was 10.3 (5.7) [0.4–48.3] for patients who did PSG1 first and 10.7 (6.1) [0.7–65.6] for those who did HSAT first (*p* = 0.65, Mann–Whitney U test).

**FIGURE 3:**
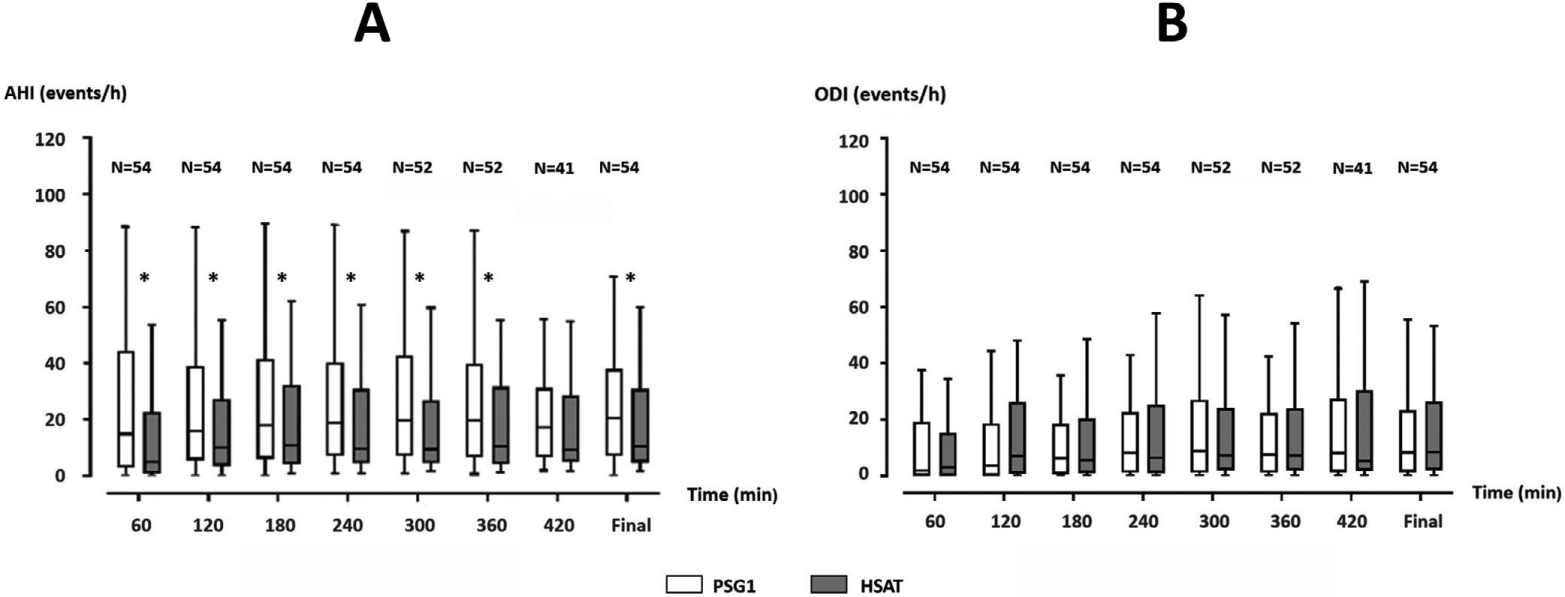
Cumulative median, interquartile range, and minimum‐maximum values in Type 1 polysomnography (PSG1) and Type 3 home sleep apnea test (HSAT) across the intervals of the night, for the apnea‐hypopnea index, AHI (A) and for the oxygen desaturation index, ODI (B). Min: Minutes; *p < 0.05 (Wilcoxon test).

**FIGURE 4:**
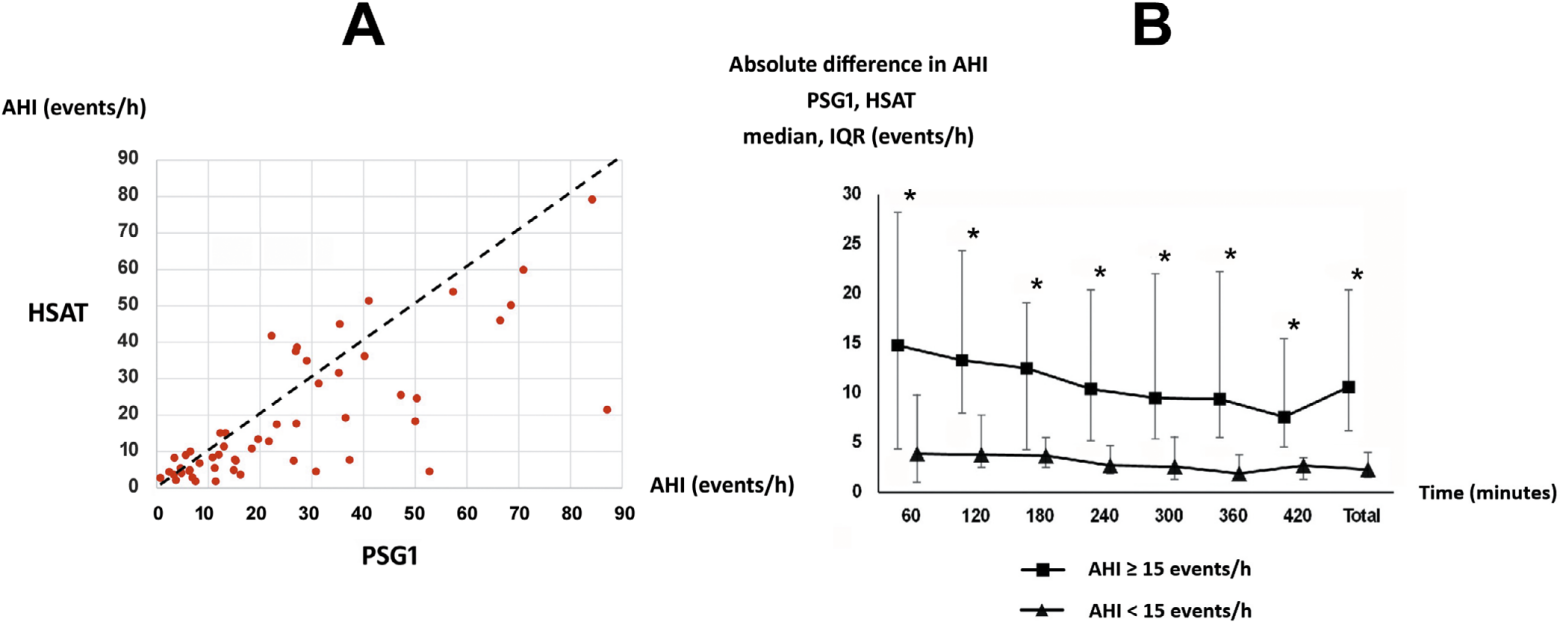
Scatter plot of the whole‐night apnea‐hypopnea index (AHI) in Type 1 polysomnography (PSG1) and Type 3 home sleep apnea test (HSAT), representing a moderate correlation (intraclass correlation coefficient = 0.70) (dashed line: Line of perfect agreement) (A). Cumulative median and interquartile range (IQR) for the absolute difference in the AHI between PSG1 and HSAT across the intervals of the night, according to the severity groups: AHI < 15 and AHI ≥ 15 events/h. *p < 0.05 between severity groups (Mann–Whitney U test) (B). [Color figure can be viewed in the online issue, which is available at www.laryngoscope.com]

When analyzed in 60‐min intervals, PSG1 exhibited a significantly higher AHI across all exam intervals, with the exception of the 420‐min interval (Figure 3A). No significant difference in ODI was observed between PSG1 and HSAT across any exam interval (Figure 3B). Agreement for the final AHI between both tests was moderate (intraclass correlation coefficient = 0.70, *p* < 0.001; Figure 4A). When patients were segregated into severity groups, those with an AHI < 15 events/h (*n* = 21) achieved and maintained an absolute difference in AHI between exams significantly below 5 events/h (a clinically irrelevant difference) from 4 h into the tests until their conclusion [mean difference for the whole night (median): 3.2 (2.3) events/h (95% confidence interval, CI: 1.5–4.0), *p* = 0.005]. Conversely, patients with an AHI ≥ 15 events/h (*n* = 33) never achieved an absolute difference in AHI between exams significantly below 5 events/h at any point [mean difference for the whole night (median): 15.7 (10.6) events/h (95% CI: 7.6–19.0), *p* = 0.999]. Furthermore, this group's absolute AHI difference never fell significantly below 15 events/h at any test interval (*p* = 0.22 for the whole‐night results). The comparison of absolute differences in AHI between these severity groups was significantly different across all test intervals (Figure 4B). With HSAT, 7/33 patients (21.2%) in the moderate‐to‐severe group were reclassified, from moderate OSA on PSG1 to mild OSA on HSAT. From 3 h onward in HSAT, 44/54 patients (81.5%) matched their PSG1 classification as simple snorer/mild OSA or moderate‐to‐severe OSA.

**FIGURE 5:**
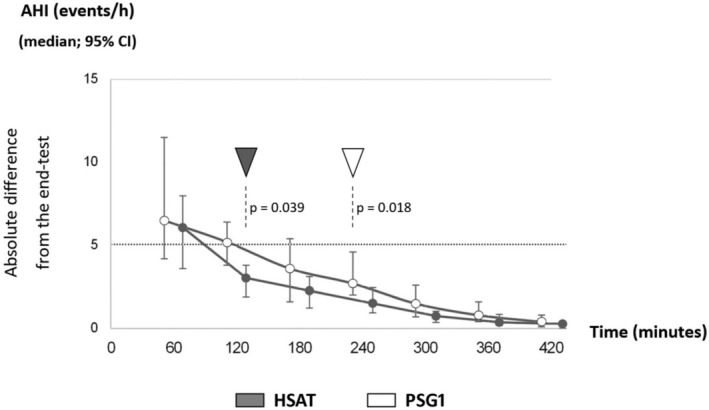
For the entire cohort, the absolute difference in the apnea‐hypopnea index (AHI) (median and 95% confidence interval, CI) between each 60‐min interval and the end‐test AHI is presented for Type 1 polysomnography (PSG1) and Type 3 home sleep apnea test (HSAT). Arrowheads indicate the minimum required recording time, defined as the point at which the test significantly achieves and maintains an AHI difference below 5 events/h, for PSG1 (white arrow, at 240 min, *p* = 0.018) and HSAT (gray arrow, 120 min, *p* = 0.039).

**FIGURE 6:**
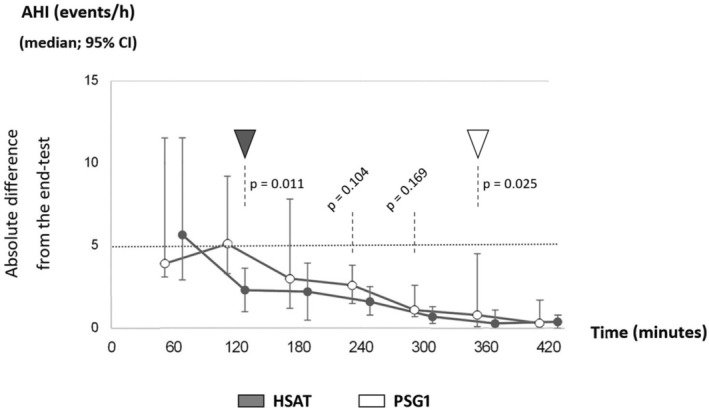
For patients with positional obstructive sleep apnea (POSA), the absolute difference in the apnea‐hypopnea index (AHI) (median and 95% confidence interval, CI) between each 60‐min interval and the end‐test AHI is presented for Type 1 polysomnography (PSG1) and Type 3 home sleep apnea test (HSAT). Arrowheads indicate the minimum required recording time, defined as the point at which the test significantly achieves and maintains an AHI difference below 5 events/h, for PSG1 (white arrow, at 360 min, *p* = 0.025) and HSAT (gray arrow, at 120 min, *p* = 0.011).

**FIGURE 7:**
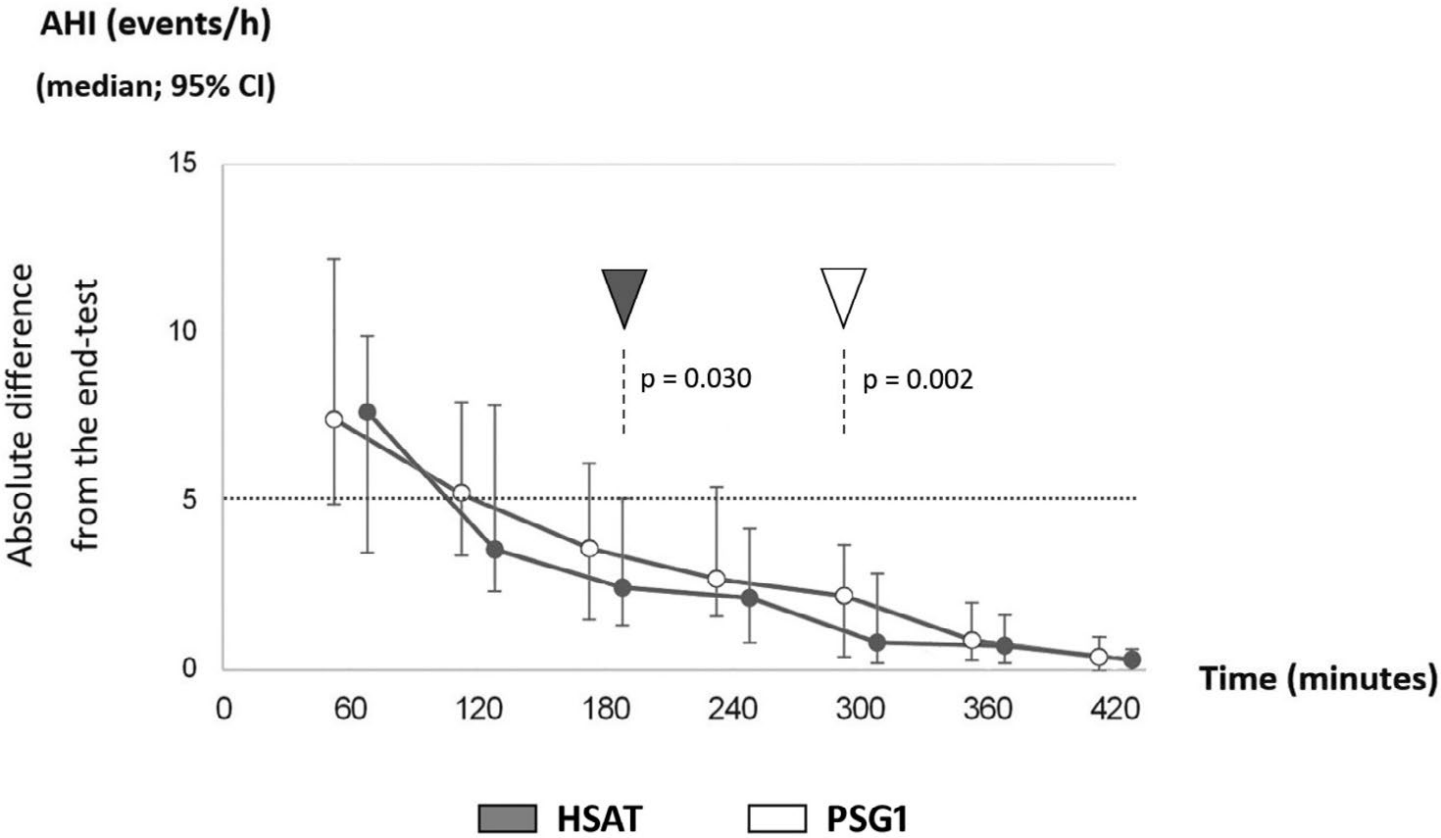
For patients with non‐positional obstructive sleep apnea (non‐POSA), the absolute difference in the apnea‐hypopnea index (AHI) (median and 95% confidence interval, CI) between each 60‐min interval and the end‐test AHI is presented for Type 1 polysomnography (PSG1) and Type 3 home sleep apnea test (HSAT). Arrowheads indicate the minimum required recording time, defined as the point at which the test significantly achieves and maintains an AHI difference below 5 events/h, for PSG1 (white arrow, at 300 min, *p* = 0.002) and HSAT (gray arrow, at 180 min, *p* = 0.030).

According to PSG1, 23 (42.6%) patients had POSA (mean AHI: 25.3 ± 17.2 events/h) and 31 (57.4%) had non‐POSA (mean AHI: 27.0 ± 25.1 events/h). For the entire cohort, the minimum RRT was 240 min in PSG1 and 120 min in HSAT (Figure 5). In the POSA subgroup, the minimum RRT values for PSG1 and HSAT were, respectively, 360 and 120 min. While the 95% CI for the median AHI difference for PSG1 in the POSA subgroup consistently fell below 5 events/h at 240 min, statistical significance for minimum RRT for PSG1 was only observed at 360 min, likely due to data variability (Figure 6). Conversely, for the non‐POSA subgroup, the minimum RRT values for PSG1 and HSAT were, respectively, 300 and 180 min (one‐sided Wilcoxon test, Figure 7).

When stratified by OSA severity, the combined simple snorer and mild OSA subgroup showed a minimum RRT value of 120 min for both PSG1 and HSAT. At this test interval (2 h), the mean (median) absolute difference from the end‐test was: 3.5 (3.3) events/h (95% CI: 1.4–4.6; *p* = 0.004) for PSG1 and 2.6 (2.1) events/h (95% CI: 0.5–3.3; *p* < 0.001) for HSAT. Conversely, for the moderate/severe OSA subgroup, minimum RRT values were 360 min for PSG1 and 240 min for HSAT (one‐sided Wilcoxon test). When applying a more relaxed threshold of 10 events/h to this subgroup, minimum RRT values decreased to 240 and 120 min for PSG1 and HSAT, respectively. At these cutoffs, the mean (median) absolute difference from the end‐test was 6.7 (4.9) events/h (95% CI: 2.7–6.5; *p* = 0.001) for PSG1 and 6.2 (3.9) events/h (95% CI: 2.3–8.0; *p* < 0.001) for HSAT.

## 4 Discussion

To our knowledge, this is the first prospective randomized crossover trial evaluating the influence of body position on minimum RRT needed to reasonably anticipate the end‐test AHI in PSG1 and HSAT. Our study revealed that HSAT achieved its minimum RRT in just 2 h for the whole cohort and POSA patients, and 3 h for non‐POSA patients. Conversely, PSG1 exhibited longer minimum RRTs, influenced by sleep position throughout the night: 4 h for the entire cohort, a minimum of 6 h for POSA patients, and 5 h for non‐POSA patients. Thus, to encompass all scenarios, our findings suggest that PSG1's minimum RRT is approximately double that of HSAT (6 vs. 3 h). While AHI was predictably higher in PSG1 than in HSAT [16] [17], ODI was similar.

Loss of monitoring signal during the night may account for up to 26% of home sleep tests requiring to be repeated [18] and knowledge on the minimum recording time for HSAT should have significant financial impact on Sleep Medicine. One previous retrospective case series reported the need of 5 h as minimum recording time for HSAT [19]. This case series reported minimum RRT based on reaching an intraclass correlation coefficient of 0.95 for AHI between the time intervals and the end‐test, a concept lacking clear clinical implications. We believe that using the difference in AHI across test intervals is more interpretable for clinical practice. Another study [20], using different analysis and defining hypopneas as a decrease in airflow > 50% (in addition to oxygen desaturation of ≥ 3%), reported that at least 390 min would be needed for AHI in HSAT to approximate AHI in PSG1. Along with likely evolution on the technology of the sensors for HSAT over the years, different criteria for hypopneas may have accounted for different outcomes in these studies. One strength of our study is the use of current definition for hypopneas [13].

Our outcomes are somewhat unexpected, because we found much shorter minimum RRT in HSAT versus PSG1 and no influence of POSA on HSAT but a major influence on PSG1. We discuss in the next paragraphs three non‐independent factors that may be responsible for our findings.

Some studies showed that patients may spend less time in supine during HSAT compared to PSG1 [21, 22] while one other did not [23]. Patients with greater AHI variations between PSG1 and HSAT also have greater variations in supine AHI and the time recorded in the supine position across the two test locations [9]. In our study, time in supine was similar in both tests. Nevertheless, sleep position represented a key variable in our findings. Compared to non‐monitored sleep, wearing polysomnography apparatus increases the amount of supine position during sleep. In both tests, we used a position sensor attached to the patient's chest. However, the position of the head in supine (and not only the trunk in supine) seems to significantly increase AHI in 30% of the patients [24]. It is feasible that, in HSAT, without head sensors, patients could move their heads to the side more easily and still keep their chest supine. This could reduce their AHI, particularly in POSA patients, thus reducing the variability of AHI influenced by position and making the outcomes of HSAT earlier predictable. To investigate this, future research should include forehead position sensors and calculate supine AHI across the night intervals.

Across the nights, there was no significant difference regarding the ODI between the tests. Despite that, AHI was higher in PSG1 in all exam intervals except at 7 h of the tests. This points to a likely role of arousal‐based hypopneas (hypopneas scored on PSG1 due to arousal rather than oxygen desaturation) in accounting for increased AHI in the laboratory, but this, per se, does not explain the higher unpredictability of end‐test AHI across PSG1. Rapid eye movement (REM) sleep occurs predominantly in the second half of the night. Patients with REM OSA (meaning the REM AHI is at least twice the non‐REM AHI) appear to have significantly higher prevalence of low respiratory arousal threshold than patients with non‐REM OSA [25]. Therefore, following the hypopnea definition rule, arousal‐based hypopneas would likely be higher at the end of the exam in patients with REM OSA, thus possibly accounting for higher AHI variability along PSG1 compared to HSAT. Future analysis of REM AHI and non‐REM AHI across the night is required to investigate this hypothesis.

The third factor that could explain our outcomes is the influence of the test‐setting (in‐laboratory versus home). In general, patients present improved sleep during sleep tests at home compared to a sleep laboratory, which means they have fewer arousals, reduced wake time, higher sleep efficiency, and a greater percentage of REM and slow‐wave sleep in HSAT compared to PSG1 [26]. One large cohort reported average total sleep time of only 5.8 h and median sleep efficiency of 79% in PSG1 [27]. This suggests that sleep consolidation occurs more rapidly at home, likely due to the burden of multiple sensors and first‐night effect discomfort in the laboratory, frequently resulting in missing the first REM period during PSG1 [28]. To our knowledge, it is currently unknown how these findings impact minimum RRT for PSG1 but, clearly, they point to a higher instability of sleep in the laboratory which predicts, as in our study, more test time in PSG1 to anticipate the end‐test AHI. Moreover, this could increase body position changes in PSG1 (a variable not measured in our study) and induce greater AHI variability across the night in the lab in patients with POSA (despite not increasing total supine time). This is a likely reason for night‐to‐night variability in AHI being lower in HSAT compared to PSG1 [29]. Based on our findings, the current recommendation of a minimum recording time of 2 h scoring an AHI ≥ 15 events/h to proceed to a CPAP titration in split night PSG1 seems inadequate to fulfill a diagnosis of moderate/severe OSA, particularly in patients with POSA [14].

We recognize there are limitations in this study. This was a single center trial and the sample size is relatively small, although the crossover design allowed a satisfactory statistical analysis. As with most HSAT devices, ours did not identify wake/sleep states, and sleep indices were based on recording time. Additionally, it did not detect arousal‐based hypopneas. These discrepancies with PSG1 account for the lower and less accurate AHI obtained with HSAT. Also, our outcomes should apply to patients with suspected OSA without relevant comorbidities (including insomnia) and cannot be generalized. Finally, we did not investigate the prevalence of REM‐dependent OSA in this study, and our conclusions may not apply to this specific population.

## 5 Conclusion

The minimum RRT for PSG1 and HSAT that safely reflect the end‐test AHI is, respectively, 6 and 3 h. Positional OSA prevents PSG1 from adequately anticipating the resulting AHI before the test is almost complete, whereas HSAT fulfills this task after 3 h of monitoring suspected OSA patients, regardless of the presence or not of positional OSA. This raises confidence in HSAT results and discomfort regarding needed monitoring time in PSG1, particularly in patients with positional OSA.

## Funding

The authors have nothing to report.

## Conflicts of Interest

Raquel Teixeira works and owns a sleep laboratory and Michel Cahali stockholder in the Brazilian company Biologix that markets a home sleep apnea test (not used in this study).

## Data Availability

The data that support the findings of this study are available on request from the corresponding author. The data are not publicly available due to privacy or ethical restrictions.
